# Functional Impacts of the Endobutton Technique in Distal Biceps Tendon Repair

**DOI:** 10.7759/cureus.108849

**Published:** 2026-05-14

**Authors:** Nuha Maryam, Neil Ashwood, Daniel Morris, Andrew P Dekker, Aiman A Ishaq, Saud Danyowi

**Affiliations:** 1 Trauma and Orthopaedics, University Hospitals of Derby and Burton NHS Foundation Trust, Burton-on-Trent, GBR; 2 Trauma and Orthopaedics, University Hospitals of Derby and Burton NHS Foundation Trust, Derby, GBR; 3 Trauma and Orthopaedic Surgery, University Hospitals of Derby and Burton NHS Foundation Trust, Burton-on-Trent, GBR

**Keywords:** distal biceps tendon (dbt) repair, distal biceps tendon repair, ortho rehabilitation, ortho-surgery, tendon reconstruction

## Abstract

Background

Distal biceps tendon rupture is an uncommon injury that can result in significant impairment of elbow flexion and forearm supination. Surgical repair is commonly recommended for active patients, with a range of fixation techniques described. While bicortical Endobutton fixation (BEF) has demonstrated favourable biomechanical properties, clinical data describing functional recovery over time remain limited.

Methods

This retrospective case series evaluated ten patients who underwent distal biceps tendon repair (DBTR) using a single-incision anterior approach with BEF. Isometric elbow flexion and radioulnar supination strength were assessed at 3 and 12 months post-operatively using a dynamometer and compared with the contralateral, uninjured limb. Post-operative complications were recorded.

Results

At three months post-operation, both flexion and supination strength of the repaired limb were significantly reduced compared with the contralateral limb. By 12 months, flexion strength demonstrated substantial recovery and was no longer significantly different from the control limb, whereas supination strength remained significantly reduced. Mean flexion strength relative to the contralateral limb improved from 68.34% to 92.77% between 3 and 12 months, while mean supination strength improved from 48.81% to 69.89%. Two (20%) patients experienced transient neuropraxia, with no tendon re-ruptures observed.

Conclusion

DBTR using a BEF technique resulted in progressive functional recovery over 12 months. Elbow flexion strength demonstrated substantial recovery within the first post-operative year, while recovery of forearm supination strength was slower and less complete. These findings may assist with rehabilitation planning and patient counselling regarding expected functional outcomes following this technique.

## Introduction

The rupture of the distal biceps tendon is an uncommon injury, [1] with over 95% of patients being male (predominantly aged between 35 and 55 years). [2] Ruptures of the distal bicep tendon are believed to occur ensuing an abrupt eccentric load on the contracted bicep, which results in a complete or partial tear [3]. Following the injury, the patient will present with weak supination of the forearm and elbow flexion [4]. Surgical repair is often the treatment for younger patients, although conservative management can also be used to treat the rupture [5] (immobilisation using a sling, superseded by physical therapy). The purpose of this study was to assess progressive recovery following distal biceps tendon repair (DBTR) through sequential isometric strength testing.

While a range of fixation techniques have been described for DBTR, including suture anchors, interference screws, and cortical button constructs, there remains variability in reported functional outcomes, particularly with respect to forearm supination strength [6,7]. Although biomechanical studies have demonstrated strong fixation properties with Endobutton techniques, fewer clinical studies have characterised the pattern of strength recovery over time following repair, especially using objective, sequential strength assessment. As a result, there remains some uncertainty regarding expected recovery timelines and the relative restoration of flexion and supination strength following this technique in routine clinical practice.

The term "biceps brachii" is derived from its two heads: the short head and the long head. The short head originates from the coracoid process via a broad, flattened tendon and lies superficial to the coracobrachialis origin. In contrast, the long head arises from the supraglenoid tubercle as a narrow, oval tendon, variably blending with the superior glenoid labrum [8].

The brachialis and biceps brachii are both flexors; however, the biceps brachii primarily functions against resistance [3]. The distal biceps tendon is made up of multiple, separate bands with an overlying tendon sheath, thereby acting as autonomous units conducting forces as individual units [4]. The left and right tendons wind in opposite directions (with the left winding clockwise and the right winding counterclockwise) [5]. Anatomical specimens have shown that the tendon is crossed on the volar aspect by a single radial recurrent artery; additionally, travelling dorsal to the tendon is an additional recurrent branch off the brachial artery that is positioned proximally to the radial recurrent artery [9,10].

 Aims and objectives

The aim of this study was to evaluate the pattern of functional recovery following DBTR using a BEF fixation technique, with particular emphasis on the differential recovery of elbow flexion and forearm supination strength over a 12-month period, as measured by sequential isometric strength testing and comparison with the contralateral limb.

## Materials and methods

This study was a retrospective, multisurgeon case series evaluating functional recovery following DBTR using a BEF technique. Ten male patients underwent surgical repair following a traumatic rupture of the distal biceps tendon between August 2018 and September 2021. The mean age at the time of surgery was 46 years (range 23-58 years). All patients were right-hand dominant; four sustained injuries to the dominant limb and six to the non-dominant limb.

Inclusion criteria were patients who underwent BEF following DBTR, had available follow-up data at 3 and 12 months, and were ≥18 years old at the time of the surgery. Exclusion criteria were non-operative management of DBTR, patients with associated injury/deficit to an extent that they could not participate with strength testing, and those lost to follow-up at either 3 or 12 months.

All procedures were performed using a single-incision anterior approach with inlay repair and BEF, resulting in reattachment of the tendon through the volar cortex of the radial tuberosity [11]. Surgical technique was consistent across cases, although procedures were performed by multiple surgeons.

Post-operative rehabilitation followed a standardised protocol across the cohort. During the first two weeks, patients were encouraged to perform passive and active-assisted range of motion (ROM) exercises within a pain-free range. From weeks 2-6, progression to active ROM was permitted, with avoidance of resisted elbow flexion and forearm supination.

From six weeks post-operatively, gradual introduction of light resistance exercises was initiated under physiotherapy supervision, with progressive loading tailored to patient tolerance. Strengthening of elbow flexion was typically introduced prior to supination-specific loading, reflecting tendon healing constraints. Return to unrestricted activity, including heavy lifting and sport, was generally permitted after 12 weeks, subject to clinical assessment. Patients who required bracing (elbow fixed-flexion ≥45°) followed a modified protocol with staged extension over six weeks before progressing to strengthening phases. Broadly similar protocols were followed through physiotherapy input, but minor variances did occur based on patient condition/ compliance.

Functional outcomes were assessed using isometric strength testing at 3 and 12 months post-operatively. Strength measurements were obtained using a BTE Primus Dynamometer (Baltimore Therapeutic Equipment Company, Baltimore, MD, USA).

Patients were positioned seated, with the shoulder adducted and neutrally rotated, elbow flexed to 90°, and forearm in either neutral (for flexion testing) or full supination (for supination testing). For each movement, patients performed three maximal voluntary contractions, each held for 3-5 seconds, with a 30-second rest interval between attempts. The highest recorded value was used for analysis. Testing was conducted bilaterally during the same session to minimise inter-session variability, with the contralateral limb serving as an internal control. All testing was carried out by suitably trained personnel.

Statistical analysis was performed using paired Student’s t-tests to compare strength values between the repaired limb and the contralateral control limb at each time point (3 and 12 months). Statistical significance was defined as P < 0.05. Changes in the percentage strength difference between limbs over time were calculated to assess the pattern of recovery.

Surgical technique

**Figure 1:**
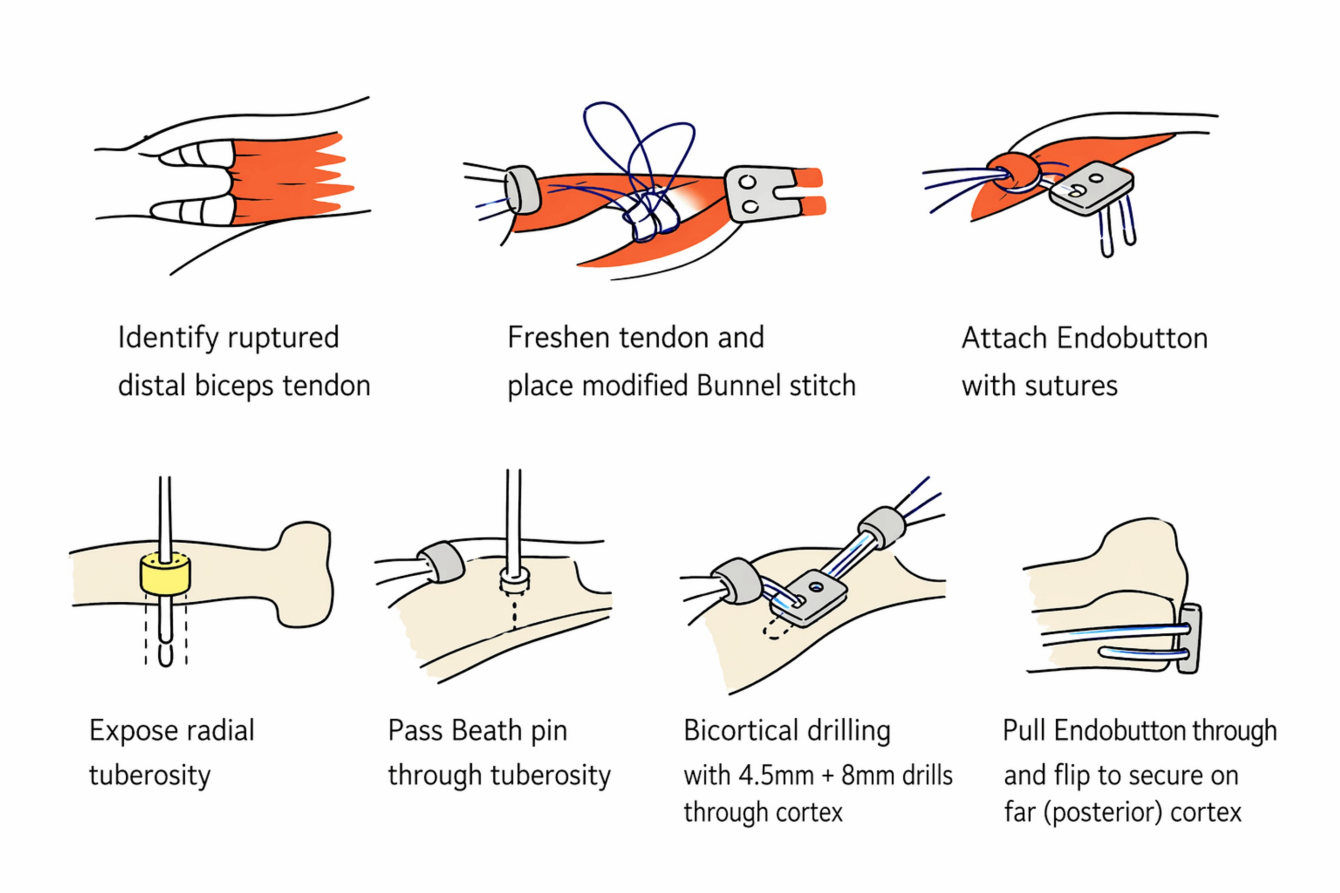
Illustration of the surgical technique Image generated using Adobe Photoshop and Inkscape

Procedures were performed under general anaesthesia or regional block according to patient factors, anaesthetic suitability, and surgeon preference. In general, general anaesthesia was selected when greater operative complexity or complete immobility was preferred, whereas regional block was used when suitable to optimise perioperative analgesia. Figure 1 displays the technique used.

An upper arm tourniquet was used, and an S-shaped anterior skin incision was made over the cubital fossa. The ruptured distal stump was identified, the end was freshened, and a modified Bunnell stitch was made using a #5 Ethibond (Ethicon Inc., Somerville, NJ, USA). An Endobutton (Arthrex Inc., Naples, FL, USA) with a 15mm loop was secured to the tendon; in chronic cases, gradual stretching of the myotendinous unit was performed to gain additional length to facilitate the reattachment of the tendon. The arm was fully extended and supinated, and a diligent dissection was done to expose the radial tuberosity, with care to avoid excess retraction to prevent injury to the posterior interosseous nerve and radial nerve with hypersupination. Next, a Beath pin was passed through the radial tuberosity as medially as possible, exiting dorsally through the skin. Bicortical drilling with a 4.5mm cannulated drill was performed, followed by an 8mm Acorn drill to prepare the hole in near cortex for incision of the tendon. A #2 Vicryl was passed through the leading hole of the Endobutton, 0 Vicryl was used for the trailing hole, and the sutures were threaded through the eyelet of the Beath pin. The Beath pin along the sutures was pulled through the dorsal aspect of the forearm with tension placed on the leading suture to deliver the Endobutton through the dorsal cortex and the tendon stump into the cortical window. The Endobutton was engaged by tensioning the trailing suture, with the elbow in slight flexion. Following this, the elbow was taken through the ROM to confirm the tendon was well attached. The wound was then closed after irrigation, and a compression bandage was applied.

Postoperative management and rehabilitation

Six patients who had elbow fixed-flexion ≥ 45° were given a brace. All patients were allowed full ROM, except for the six patients who were given a brace (in which case progressive permitted extension was utilised for six weeks).

## Results

**Figure 2:**
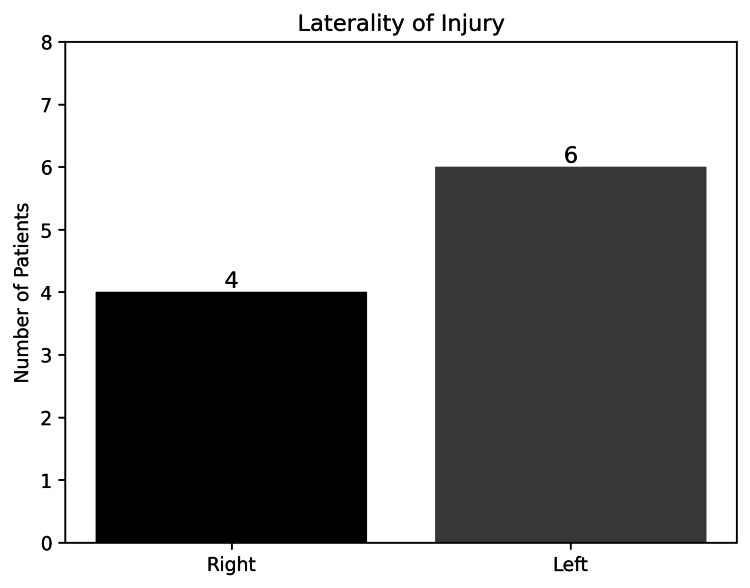
Laterality of injury

**Figure 3:**
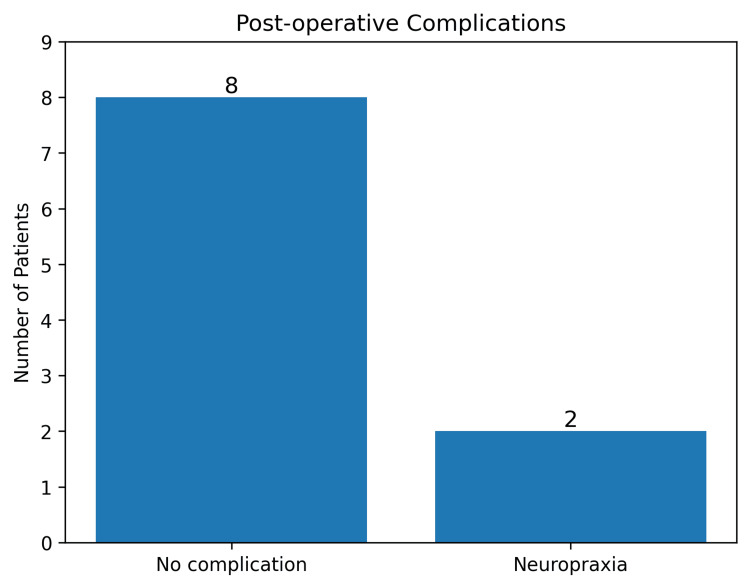
Post-operative complications following distal biceps tendon repair

Altogether, 10 DBTRs were included in the evaluation. Figure 2 shows how many patients were right- or left-handed, while the mean patient age was 45.9 years (with a range of 23-58 years and a median of 50 years). Overall, the DBTR of two (20%) patients resulted in complications (lateral cutaneous nerve of the forearm neuropraxia and posterior interosseous nerve neuropraxia). Post-operative complications observed in this cohort are summarised in Figure 3. Flexion strength at 12 months was non-significant (P = 0.063), whereas flexion strength at three months (P = 0.012), supination strength at three months (P = 0.005), and supination strength at 12 months (P = 0.041) were all significant.

**Figure 4:**
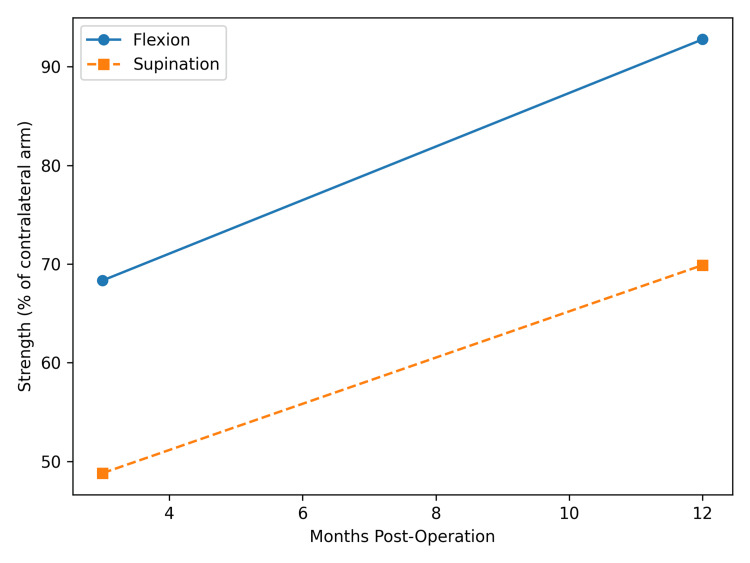
Recovery of flexion and supination strength over time

**Figure 5:**
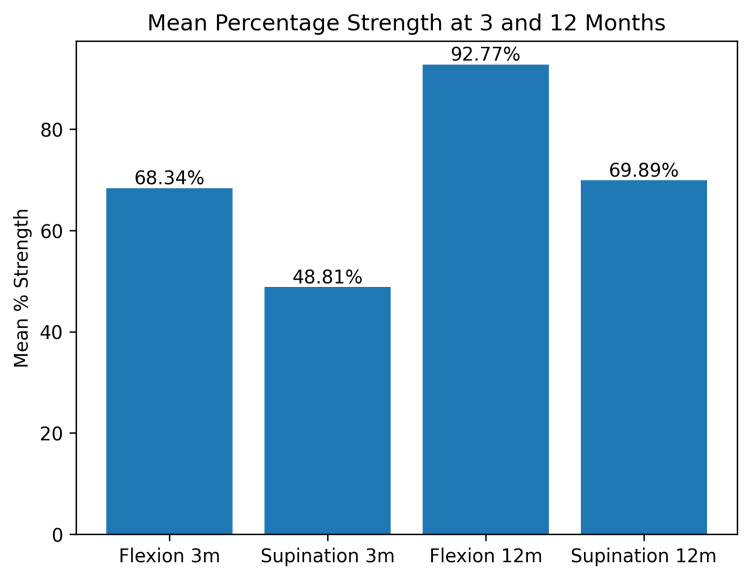
Mean percentage strength at 3 and 12 months

**Figure 6:**
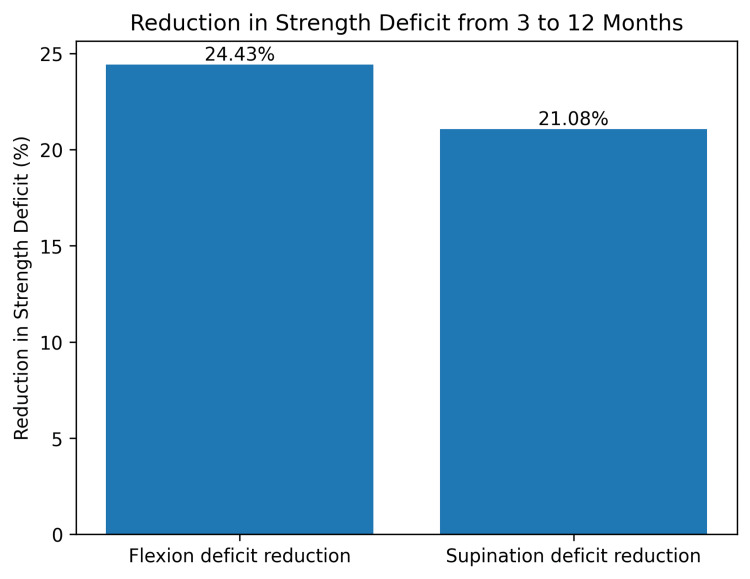
Reduction in strength deficit from 3 to 12 months

Mean flexion strength was 68.34% at three months and improved to 92.77% at 12 months, while mean supination strength improved from 48.81% to 69.89% over the same period. At 12 months, mean flexion strength was 92.77% (SD 10.74), while mean supination strength was 69.89% (SD 20.34) relative to the contralateral limb. The overall pattern of recovery in elbow flexion and forearm supination strength over time is illustrated in Figure 4. The mean percentage strength values for flexion and supination at 3 and 12 months are summarised in Figure 5. There was a 24.43% decrease in the mean % difference in flexion strength between the control and repair arm and a 21.08% decrease in the mean % difference in supination strength between the control and repair arm over nine months. The reduction in strength deficit between the repaired and contralateral limbs from 3 to 12 months is illustrated in Figure 6.

## Discussion

Comparison with existing literature

The pattern of recovery observed in this study is broadly consistent with previously published literature on DBTR. Several studies have reported that elbow flexion strength tends to recover more reliably and earlier than forearm supination strength following surgical repair, regardless of fixation method [11-17]. Nesterenko et al. demonstrated persistent deficits in supination strength following distal biceps rupture, even after surgical intervention, highlighting the difficulty in fully restoring rotational strength of the forearm [11]. Similar findings have been reported in anatomical and biomechanical studies examining the complex insertional anatomy of the distal biceps tendon [12,15].

With respect to fixation technique, BEF has been shown in biomechanical studies to provide strong initial fixation and resistance to failure, which may facilitate early mobilisation and functional recovery. Jarrett et al. [1] highlighted the distinct contributions of the short and long head components of the distal biceps tendon, suggesting that restoration of native tendon orientation may be critical for optimal supination strength. In this context, the persistent supination deficit observed at 12 months in the present study is not unexpected and is consistent with prior reports.

Reported complication rates following DBTR vary across the literature, with transient neuropraxia being among the most commonly described complications. The 20% complication rate observed in this study, consisting of transient lateral cutaneous nerve of the forearm neuropraxia and posterior interosseous nerve neuropraxia, is comparable to rates reported in other series utilising single-incision techniques [14,17]. Importantly, no tendon re-ruptures were observed, which is in keeping with studies suggesting low re-rupture rates following Endobutton fixation.

Overall, the findings of this study align with existing evidence suggesting that DBTR using an Endobutton technique provides reliable restoration of elbow flexion strength, while recovery of supination strength may be more variable and prolonged [18].

Interpretation of results

The difference in recovery between flexion and supination strength is likely related to the biomechanical function of the distal biceps tendon. While reattachment of the tendon effectively restores elbow flexion, achieving full restoration of supination strength may be more challenging due to factors such as tendon orientation, fixation position, and the complex rotational mechanics of the forearm. The relatively large standard deviation observed in flexion strength suggests considerable inter-patient variability in recovery, whereas supination strength demonstrated a more consistent pattern across the cohort.

In addition, early post-operative rehabilitation may place greater emphasis on flexion-based activities, with supination strength addressed later in the recovery process. Protective loading strategies and patient apprehension in the early stages of rehabilitation may further contribute to delayed recovery of rotational strength.

Clinical implications

From a clinical perspective, these findings are relevant for both rehabilitation planning and patient counselling. Patients can be advised that meaningful recovery of elbow flexion strength is likely within the first year following surgery. However, residual deficits in supination strength may persist at 12 months, particularly in patients with higher functional demands.

Rehabilitation protocols may benefit from targeted supination strengthening once tendon healing allows, particularly for patients aiming to return to manual work or sporting activities that place greater demand on forearm rotation.

Limitations

The findings of this study should be interpreted in the context of its limitations. The small sample size and retrospective design limit the strength of the conclusions that can be drawn. Additionally, the multisurgeon nature of the study introduces potential variability in surgical technique. Larger, prospective studies with longer follow-up would be useful to further define the recovery timeline, particularly for supination strength beyond 12 months. A further limitation is the use of the contralateral limb as a control. Strength discrepancy between dominant and non-dominant hands can influence percentage strength recovery. As a result, recovery outcomes may be slightly over-reported in the non-dominant group.

## Conclusions

In this small retrospective case series population, DBTR using a BEF technique resulted in generally favourable functional outcomes in this cohort. Progressive recovery of both elbow flexion and forearm supination strength was observed over the 12-month post-operative period. By 12 months, flexion strength had largely recovered when compared with the contralateral limb, whereas supination strength remained reduced, indicating a slower and less complete recovery of forearm rotational strength. These findings suggest that the Endobutton technique is effective in restoring elbow flexion following acute distal biceps tendon rupture, while recovery of supination strength may continue beyond the first post-operative year.

This may have practical implications for rehabilitation planning and patient counselling, particularly for individuals whose work or sporting activities place higher demands on forearm rotation. While the results of this study are encouraging, they should be interpreted with consideration of the study’s limitations, including the small sample size and retrospective design. Further prospective studies with larger patient cohorts and longer follow-up are required to better define the recovery profile following DBTR.
